# Scaling the U-net: segmentation of biodegradable bone implants in high-resolution synchrotron radiation microtomograms

**DOI:** 10.1038/s41598-021-03542-y

**Published:** 2021-12-20

**Authors:** Ivo M. Baltruschat, Hanna Ćwieka, Diana Krüger, Berit Zeller-Plumhoff, Frank Schlünzen, Regine Willumeit-Römer, Julian Moosmann, Philipp Heuser

**Affiliations:** 1 Deutsches Elektronen-Synchrotron DESY, Notkestr. 85, 22607 Hamburg, Germany; 2 Institute of Metallic Biomaterials, Helmholtz-Zentrum Hereon GmbH, 21502 Geesthacht, Germany; 3 Institute of Materials Physics, Helmholtz-Zentrum Hereon GmbH, 21502 Geesthacht, Germany; 4 Helmholtz Imaging, Deutsches Elektronen-Synchrotron DESY, Notkestr. 85, 22607 Hamburg, Germany

**Keywords:** Materials science, Structural materials, Image processing

## Abstract

Highly accurate segmentation of large 3D volumes is a demanding task. Challenging applications like the segmentation of synchrotron radiation microtomograms (SRμCT) at high-resolution, which suffer from low contrast, high spatial variability and measurement artifacts, readily exceed the capacities of conventional segmentation methods, including the manual segmentation by human experts. The quantitative characterization of the osseointegration and spatio-temporal biodegradation process of bone implants requires reliable, and very precise segmentation. We investigated the scaling of 2D U-net for high resolution grayscale volumes by three crucial model hyper-parameters (i.e., the model width, depth, and input size). To leverage the 3D information of high-resolution SRμCT, common three axes prediction fusing is extended, investigating the effect of adding more than three axes prediction. In a systematic evaluation we compare the performance of scaling the U-net by intersection over union (IoU) and quantitative measurements of osseointegration and degradation parameters. Overall, we observe that a compound scaling of the U-net and multi-axes prediction fusing with soft voting yields the highest IoU for the class “degradation layer”. Finally, the quantitative analysis showed that the parameters calculated with model segmentation deviated less from the high quality results than those obtained by a semi-automatic segmentation method.

## Introduction

Magnesium (Mg) and its alloys are highly attractive as temporary implant materials due to their good biocompatibility and biodegradability^1^. Mg-based materials have sufficient initial strength for load-bearing applications and degrade under physiological conditions in products that are well-tolerated by the body, avoiding the need for a second surgical intervention to remove the implant after bone healing^1^. A major challenge is tailoring the degradation in a manner that is suitable for a biological environment. Fast or uncontrolled corrosion is associated with strong hydrogen and ion release and severe pH changes, which can lead to a fast loss of mechanical stability and undesirable biological reactions^2^. In order to characterize Mg bone implants and the associated degradation process, numerous imaging experiments are being conducted including in situ loading experiments^3–5^, corrosion experiments^6,7^ and long-term studies at the micro- and nanometer scale. *In situ* measurements and the high amount of samples to be scanned at a high spatial resolution require the use of synchrotron radiation microtomography (SRμCT). At the same time, SRμCT enables high-throughput experiments, which are required to image a sufficiently large number of samples to achieve statistical power in animal experiments.

To gain quantitative information about the material degradation and bone regeneration, SRμCT images need to be analyzed. Therefore, we perform a semantic segmentation of images i.e., the partitioning of pixels or voxels into segments (labels). In this study, we analyze explants containing the degraded implant and surrounding bone, and are interested in creating labels representing residual material (RM) and degradation layer (DL) of the implant, and bone. However, common segmentation approaches (thresholding, watershed^8^, WEKA^9^) fail due to the high textural variation of the corroded areas. These areas are highly fragmented and exhibit a high variation in electron density resulting in grayscale values that vary about the value of the residual material and that finally approach the grayscale level of bone (see Supplementary Fig. S1). Moreover, the segmentation task is further aggravated by small phase contrast-induced edge enhancement in the reconstructed volumes which is due to the high coherence at the tomography end station and a non-vanishing propagation distance between sample and detector system.

In the last decade, deep learning has replaced classical methods for problem solving in many fields. Especially in computer vision and medical image processing, deep learning surpasses classical methods^10^. Fully convolutional neural networks (FCNs)^11^ were proposed early on for dense semantic segmentation. The basic architecture of FCNs can be described by an encoder and decoder path. Long et al.^11^ fused the information of different decoder scales by summation to get a finer segmentation result. Ronneberger et al.^12^ was one of the first to adapt FCNs for medical image segmentation and proposed the U-net architecture. Other than the FCN, the U-net contains skip-connections from encoder to decoder path. To fuse the information of different scales, a concatenation approach is used (state-of-the-art results for the ISBI cell tracking challenge 2015^13^).

Medical image data frequently are 3D volumetric data, and exploiting the full 3D context using 3D convolutions as proposed by Milletari et al.^14^ would certainly be beneficial for some applications. However, in 3D-FCNs the number of parameters increases with a power of three, which quickly can become an intractable problem, and greatly increases the risk of over-fitting particularly for small training datasets, which is inevitably the case for 3D datasets. Finally, processing of large volumes (e.g. $512 \times 512 \times 512$ voxels) is still not possible due to limited GPU memory on currently available hardware. For semantic segmentation in high-resolution SRμCT volumes, both these problems of small training datasets and over-fitting need to be addressed carefully. Prior work on semantic segmentation of biodegradable bone implants in SRμCT, focused on training a 3D-FCN with limited amount of annotation data^15^. While Bockelmann et al. showed first promising results, the dice-score for the label “corroded screw” is 0.541 and the overall segmentation results are not sufficient for a quantitative analysis without major manual corrections.

**Figure 1:**
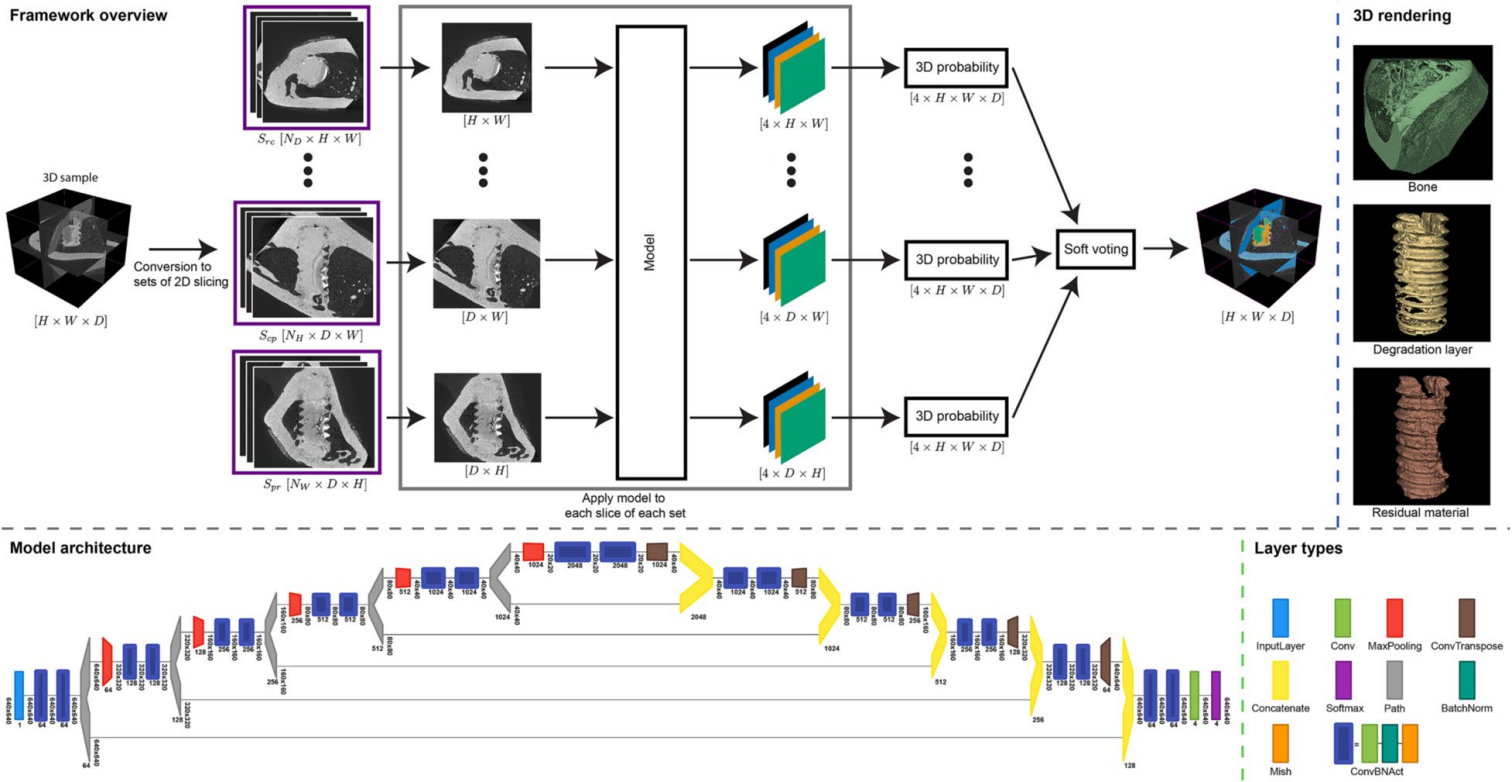
Overview of the segmentation framework for high-resolution synchrotron radiation microtomograms. Top shows the full segmentation pipeline: 1. conversion of 3D tomograms into 2D slices. 2. processing of slicing sets by our model. 3. soft voting is used to fuse the multi-axes prediction into the final segmentation. Top right: 3D rendering of the resulting segmentation (created with 3D Slicer, v4.11, https://www.slicer.org/). Bottom shows our final U-net model architecture and the layer legend (created with Net2Vis^38^, https://github.com/viscom-ulm/Net2Vis).

In this work, we focus on the development of a fully automatic segmentation framework (see Fig. 1) for SRμCT volumes. We are evaluating the method for the specific use case of characterizing biodegradable bone implants, but it is also suitable for other use cases after retraining. For our use case, we provided not only state-of-the-art segmentation results but also show that the obtained segmentation allow a quantitative analysis. We perform a systematic evaluation using 4-fold cross-validation and analyze several design decision for dense segmentation in SRμCT volumes. The paper is structured as follows: section “Methods” presents the current semi-automatic segmentation workflow and explains the different evaluated design decision of the segmentation framework. In section “Experiments and results”, we show the experimental setup and the evaluation results. Section “Discussion and conclusion” is devoted to the discussion of our results.

## Methods

In this study we used SRμCT data from 17 samples, i.e. 14 for training and validation and three for testing. The corresponding SRμCT data was acquired at the P05 imaging beamline (IBL)^16^ at PETRA III at the Deutsches Elektronen-Synchrotron (DESY) or at the Diamond Manchester Imaging Branchline I13-2 at the Diamond Light Source (I13)^17^. Depending on the type of samples, the available instrumentation, or due to technical issues, different experimental settings were used. Table 1 shows the dataset characteristics.

At IBL a monochromatic beam was used with energies ranging from 25 to 46 keV. An indirect detector system was used with a scintillator made of cadmium tungstate (CdWO4) converting X-rays to optical light which was further magnified with a $5\times$ or $10\times$ objective and then detected by a CCD or CMOS camera. The CCD camera has $3056 \times 3056$ pixels, a linear pixel size of 12 μm, a dynamic range of 16-bit and the CMOS camera has $5120 \times 3840$ pixels, a linear pixel size of 6.4 μm, and a dynamic range of 12-bit^18^. Tomograms were reconstructed using a MATLAB based framework^19,20^ and employing the ASTRA toolbox for tomographic backprojection^21,22^. At I13 a pink beam with a mean energy of 23 keV to 24 keV was used. The indirect camera system consisted of a 1.25$\times$ objective lense with a pco.edge 5.5 camera (PCO AG, Kelheim, Germany) with $2160 \times 2560$ pixel, a linear pixel size of 6.5 μm, and a dynamic range of 16-bit. The tomograms were reconstructed using the open-source Savu framework^23^ with the TomoPy reconstruction package^24^. Our reconstructed tomograms have an isotropic voxel size of 2.4 μm or 1.2 μm and spatial dimension of $2510 \times 2510 \times 2130$ voxels.

Finally, each sample was preprocessed by resampling with bi-linear interpolation to a fixed voxel size of 5 μm, clipping the dynamic range to the 0.5% and 99.9% percentile, and linearly normalizing the gray values to the range [0, 1].

### Segmentation of synchrotron radiation microtomograms

Currently, a time consuming semi-automatic workflow (WF segmentation) is needed to segment each sample into four classes: “background” (BG), “bone”, “degradation layer”, and “residual material”. The class “background” also contains the soft tissue, since it is not of interest for our questions.

The WF segmentation was performed with the use of Avizo 9.4.0 (FEI SAS, Thermo Scientific, France). We used a reference screw, lab-μCT of a preimplantation screw and SRμCT of a postimplantation screw (explant). Both μCTs were preprocessed by registration with the reference screw to align the implant vertically in the 3D volume and resampling to the fixed voxel size of 5 μm. Each segmentation of an entire SRμCT took about four days for the WF method. A detailed procedure for workflow segmentation can be found in supplementary section “Workflow segmentation”. The WF segmentation was the basis for training our machine learning segmentation framework (ML segmentation). For evaluation, a high quality segmentation (HQ) was prepared, manually correcting three additional samples—named samples 1 to 3. This manually corrected segmentation is very time-consuming (i.e., 10 to 14 days for an entire SRμCT) but delivers reliable information. All three samples were screws made of alloy Mg-5Gd implanted into a rat.

Based on the segmentation, quantitative parameters describing implant degradation and osseointegration can be obtained. In the analysis (see section “Experiments and results”), we investigate following parameters: degradation rate (DR) [mm/year], bone to implant contact (BIC) [%] and bone volume to total volume (BV/TV) [%]. The DR is calculated based on the volume loss of the material in relation to its initial surface area and the implantation time. We used the simplified equation from Eshwara et al.^25^:

1 $$\begin{aligned} \text {DR} = \frac{v_i-v_r}{a_i}*t, \end{aligned}$$

where $v_{i}$ and $v_{r}$ are initial and residual volume of the screw, respectively, $a_{i}$ is the initial surface area (i.e., surface of the screw before implantation) and *t* is the time of degradation.

BIC is a parameter describing how much of the degraded implant is in contact with mineralized bone and gives information about the osseointegration and the stability of the whole system^26^. The percentage of BIC is quantified by dividing the surface of the contact area by the surface area of the implant:

2 $$\begin{aligned} \text {BIC} = \frac{b}{a}, \end{aligned}$$

where *b* is the total number of boundary voxels of the implant (i.e., degradation layer and residual material combined) that are in contact with “bone”. *a* is the surface of the implant.

Finally, BV/TV delivers information about the relative bone volume in the region close to the degraded implant^27,28^. We quantify this parameter by dividing the bone volume by the total volume excluding the degradation layer in a selected distance around the implant:

3 $$\begin{aligned} \frac{\text {BV}}{{\text {TV}}} =\frac{v_{{\rm bone}}}{v_{{\rm ROI}}}, \end{aligned}$$

where $v_{{\rm bone}}$ is the total number of bone voxel in a region of interest (ROI) around the implant and $v_{{\rm ROI}}$ is the total voxel count of this ROI. BV/TV enables studying the bone content and bone regeneration over time.

### U-net model width, depth, and input size

Tan et al.^29^ showed with the EfficientNet for a classification task that the hyper-parameters model width *c* (i.e., number of channels), depth *d* (i.e., number of layers) and input size are strongly related and should be changed together to achieve the best results. Here, we adopt this approach for the segmentation task at hand and test it for the 2D U-net architecture.

Our baseline is a U-net model with some minor changes to recent advances in the field of deep learning. First, we changed all convolutional-layers (conv-layers) to use the “same” mode (i.e., automatic zero padding), so that the spatial dimensions are not reduced by the convolution. Secondly, we added batch normalization^30^ (BN) after all conv-layers and, thirdly, we changed the activation function to Mish^31^. Supplementary Tables S1a and S1b summarize our encoder and decoder structure for the baseline model, respectively.

The model width can be described as the number of output channels $c_l$ that the *l*’th conv-layer has, where *l* is often the last conv-layer of the encoder. The intuition for a wider model is that the model should be able to learn more subtle features. Here, we selected the first conv-layer $l=0$ with output channels $c_0$ to describe the model width. This is because the output channels $c_{l+1}$ of all subsequent conv-layers $l+1$ are then set to $c_{l+1} = 2 * c_{l}$. To investigate the effects of different model widths, we therefore selected different values for $c_0 \in \{32,64,96,112\}$.

The depth of a model usually refers to the total number of conv-layers the model has. Many works demonstrate that the model’s depth is an important hyper-parameter^32^. A larger depth has two desired properties. First, the receptive field^33^ of the model is increased and, secondly, more complex features can be extracted from the input image. Since the U-net model is symmetric by design (i.e., the encoder has the same number of conv-blocks as the decoder), we defined the depth by the number of conv-blocks the encoder has. While, each conv-block consists of two conv-layers for the standard U-net. For our experiments with high-resolution volumes, a larger receptive field can be very important to capture enough contextual information. Hence, we evaluated different depths $d \in \{3, 4, 5, 6\}$ with an receptive field of 188, 460, 1084, and 2492 pixels, respectively.

The input size ($\text {IS}$) with $w \times h$ is very important, as it controls the effective resolution and contextual information for the model. A small input size in combination with bi-linear downsampling considerably reduces the resolution of high dimensional data. Hence, the model cannot extract fine-grained features anymore. Even though downsampling of the images would decrease the required computational time, the high-resolution is one of the key features of the SRμCT data, and is consequently not used here. Alternatively we used random patches of the 2D slices for training and applied the model at testing to the full 2D slice. Here, a lager input size at training provides more information to the model which can be beneficial to extract complex features. In the context of semantic segmentation of SRμCT volumes with a spatial size of $1200 \times 1200 \times 1000$ voxels, we investigated three different input sizes $\{384 \times 384, 512 \times 512, 640 \times 640 \}$ pixels.

To conclude the scaling, we tested all combination (i.e., 48 different models) of the three hyper-parameters *c*, *d*, and input size.

### Incorporation of 3D information by multi-axes prediction fusing

Reliable 3D semantic segmentation in high-resolution volumes like SRμCT is still an unsolved challenge. In deep learning, different approaches currently exist to leverage the 3D information. The naive choice would be a 3D-FCN like the V-net^14^ but for small datasets and high-resolution volumes such model architecture is not feasible. Similar to Zhou et al.^34^, we process each 3D volume slice-by-slice with a 2D U-net model, while extending the idea to more than three slices. When training the model we used three sets of 2D slices, and when testing (or inference) we used three or nine sets of 2D slices.

**Figure 2:**
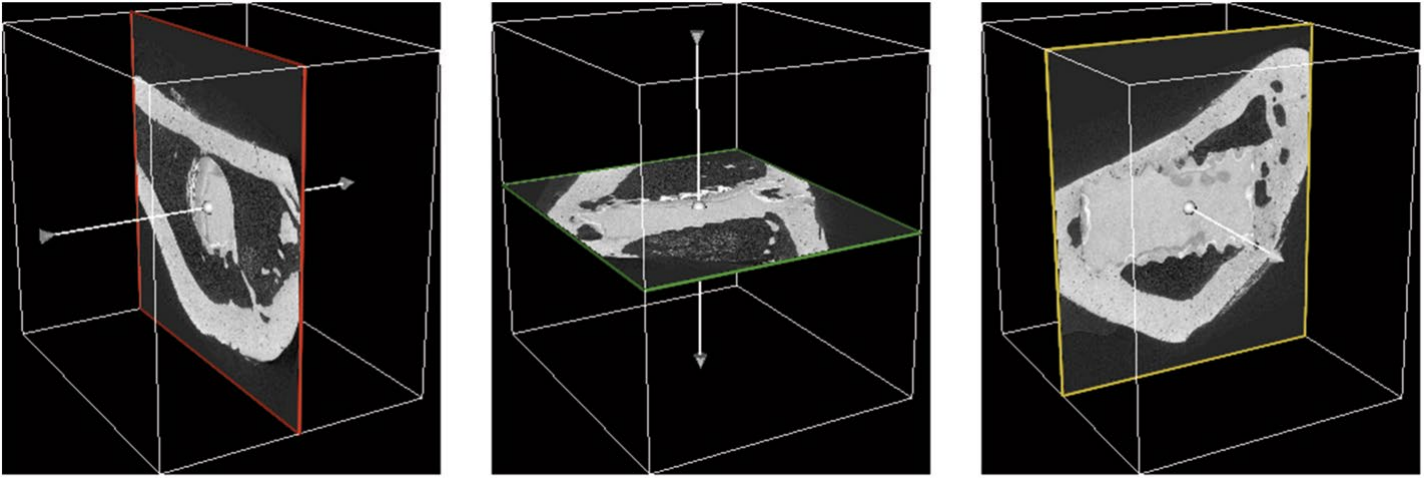
Slicing example (sample 1) for three naive planes (created with 3D Slicer, v4.11, https://www.slicer.org/). From left to right: $S_{rc}$ is show in red, $S_{cp}$ in green, and $S_{pr}$ in yellow. The arrow indicates the slicing direction.

Let ${\mathbf {V}} \in {\mathbb {R}}^{H \times W \times D}$ be our 3D volume, where *H*, *W*, and *D* are the height, width, and depth of the volume, respectively. Furthermore, *V*(*p*, *r*, *c*) is a single voxel at the location (*p*, *r*, *c*). Then, $S = \{{\mathbf {S}}_0, {\mathbf {S}}_1, \ldots , {\mathbf {S}}_N\}$ is a set of slices created from ${\mathbf {V}}$, where ${\mathbf {S}}_n \in {\mathbb {R}}^{{\hat{H}} \times {\hat{W}}}$ is a single slice and *N* is the total number of slices. Naively, we defined three sets of slicing $S_{rc}$, $S_{cp}$, and $S_{pr}$, where each set is defined by the sliced plane, e.g., ${\mathbf {S}}_n \in S_{rc}$ is defined by $S_n(i,j) = V(n,i,j)$, where $n = \{0,1,\ldots , D\}$. While, ${\mathbf {S}}_n \in S_{cp}$ and ${\mathbf {S}}_n \in S_{pr}$ are defined by $S_n(i,j) = V(i,n,j)$ and $S_n(i,j) = V(i,j,n)$, with $n = \{0,1,\ldots , H\}$ and $n = \{0,1,\ldots , W\}$, respectively. Figure 2 shows one example for each of the slicing planes.

Additionally, we proposed to include more than three slicing planes by rotating the volume around each axis (i.e. x-, y-, z-axis) and do additional slice-by-slice processing. For our experiments, we rotated the volume three times by $45^{\circ }$ along each axis. Such a rotation requires an interpolation method, a new pixel fill method, and the definition from the rotation point. Here, we used bi-linear interpolation, constant fill with zeros, and the image center, respectively. Also, the rotated volume is not cropped to the original size and is therefore larger. After the rotation, we sliced each of the three rotated volumes again using the same approach as before, resulting in nine additional slicing planes. Three of those nine slicing planes are not introducing additional information, because they are similar to the naive planes and only rotated by $45^{\circ }$. Therefore, we only used six non-redundant slicing planes and thus had in total nine slicing planes (because of the three naive planes).

Now, we employed the model to a slicing set, which resulted in a prediction ${\mathbf {P}} \in {\mathbb {R}}_{\ge 0}^{H \times W \times D \times k}$ of ${\mathbf {V}}$ (after stacking the slices back to a volume) where *k* is the number of classes to segment. To utilize the 3D information, we tested two different methods to combine the segmentation of all slicing sets. First, we considered probability averaging (also know as soft voting). Here, the combined probability:

4 $$\begin{aligned} {\mathbf {P}}_{{\rm avr}} = \dfrac{1}{M} \sum _{i=1}^M {\mathbf {P}}_{i}, \end{aligned}$$

where *M* is the number of predictions to average and ${\mathbf {P}}_{i}$ are the independent predictions.

Afterwards, we obtained the final segmentation ${\mathbf {P}}_{{\rm avr}}^{{\rm seg}} \in {\mathbb {N}}^{H \times W \times D}$ by assigning the label for the class with the highest probability. Soft voting helps to favor predictions with a high probability against low probabilities. Secondly, we employed majority voting (MV), where each prediction is equally weighted. For MV, each ${\mathbf {P}}_i$ is first converted to a segmentation ${\mathbf {P}}_i^{{\rm seg}} \in {\mathbb {N}}^{H \times W \times D}$ by the same method as in soft voting (i.e., selecting the label for the class with the highest probability). Next, the final segmentation is calculated by:

5 $$\begin{aligned} {\mathbf {P}}_{\mathrm{MV}}^{{\rm seg}} = \text {mode}\{{\mathbf {P}}_0^{{\rm seg}},{\mathbf {P}}_1^{{\rm seg}}, \ldots , {\mathbf {P}}_M^{{\rm seg}}\}. \end{aligned}$$

In other words, at each pixel the class that receives the largest number of classification (or votes) is assigned as final segmentation label.

## Experiments and results

For an assessment of the generalization performance, we performed a 4-fold cross-validation^35^ with our 14 training samples (i.e., samples with WF segmentation) and calculated our final results on three extra HQ test samples (i.e., samples with extensive and time-consuming manual segmentation). The experiments were evaluated in two steps. First, we analyzed our results using the intersection over union metric (IoU):

6 $$\begin{aligned} \text {IoU} = \frac{{\text {TP}}}{{{\text {TP}} + \text{FP} + \text {FN}}}, \end{aligned}$$

where TP are the true positives, FP are the false positives, and FN are the false negatives.

Secondly, we further evaluated the best performing model by calculating the key measures (see section “Methods”) for our segmentation and visually inspecting the segmentation.

### Implementation

To have a fair comparison between the experiments, we had a fixed training setup. Each model was trained for $1.5 \times 10^6$ iterations or till no improvement on the validation loss is noted (i.e., early stopping). We used common online data augmentation methods^36^ to extend our training dataset. When training, we sampled random patches with 85 % to 100 % of the image area and evenly distributed aspect ratios between 3 : 4 and 4 : 3. Each patch was then resized to the specific training patch size of the experiment (i.e., $384 \times 384$, $512 \times 512$, or $640 \times 640$). Furthermore, we used random horizontal flipping, random rotations between -90$^{\circ }$ to 90$^{\circ }$, random elastic deformations, random brightness and contrast changes. For validation, we only used the center crop with an size of $992 \times 992$ (i.e., no resizing). The final testing was done on the full image without any cropping and resizing. We optimized all models using ADAM^37^ and set $\beta _1$ and $\beta _2$ to 0.9 and 0.999, respectively. As loss function, we employed cross-entropy. The learning rate was set to $\text {lr}=0.0003$. While training, we reduced the learning rate by a factor of 2 when the validation loss did not improve for $10^4$ iterations. Due to model architecture variations, we used global batch sizes of 32 and 16 for the smaller and larger models, respectively. The models were implemented in Tensorflow 2.4, trained with automatic mixed precision and with data parallelism on nodes containing four Nvidia Tesla V100-SXM2-32GB. For a full overview of the implementation, our code is publicly available at https://gitlab.desy.de/helmholtz-imaging/scaling_the_u-net.

### U-net model scaling for SRμCT

**Figure 3:**
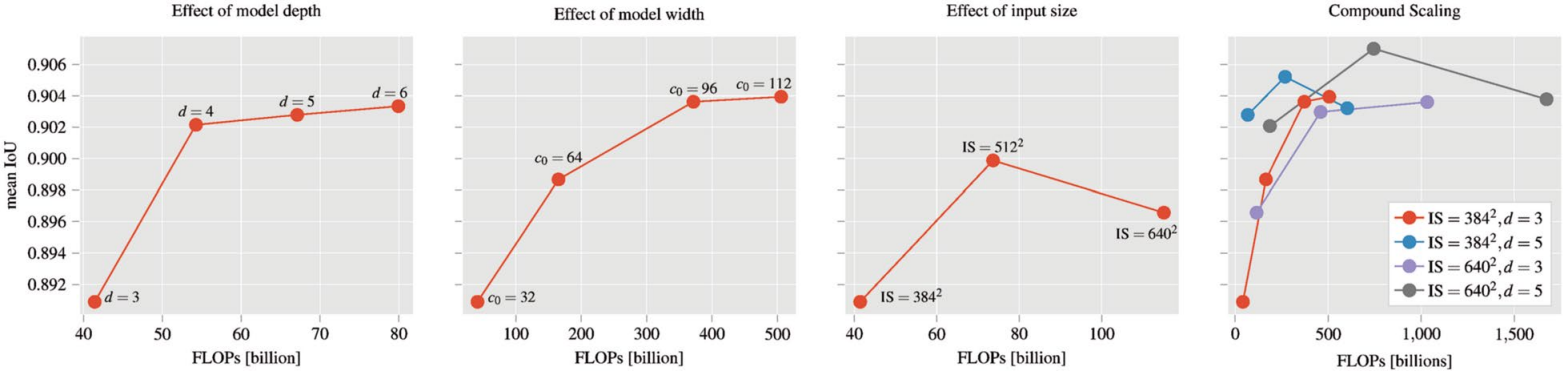
Results for scaling of the U-net. Each plot shows the mean IoU (i.e., averaged over the 4-fold cross validation and the classes) vs the floating point operations per second (FLOPs). For compound scaling, we vary the model width for each step of each run. From left to right: model depth *d*, model width $c_o$, model input size *IS*, compound scaling of multiple hyper-parameters.

Figure 3 summarizes the results for scaling each parameter of the baseline model separately (i.e., depth, width, and input size) and for scaling multiple parameters simultaneously (i.e., compound scaling). We observe that increasing the depth and width improves the mean IoU. For the depth parameter, the mean IoU increased from 0.891 to 0.903 and for the model width, the mean IoU increased to 0.904. Both show a steady increase, but with diminishing returns as the parameter is further increased. Changing the input size shows a different effect. Here, the mean IoU first increases from to 0.891 to 0.900 but then decreases again to 0.897.

For compound scaling, each plot shows a specific setup with a fixed input size and model depth (e.g., “$\text {IS}=384^2$, $d=3$” is the baseline model with input size $384\times 384$ and depth $d=3$). Only the width is varied for the different results. We notice that changing the model depth *d* and input size together with the model width *c* results in the best mean IoU of 0.906 (i.e., $\text {IS}=640 ^2$, $d=5$ and $c_0=64$). On the other hand, changing one parameter such as the depth *d* or the input size, the mean IoU only increases to a maximum of 0.905 and 0.904, respectively. For the two very large models (i.e., “$\text {IS}=384^2$, $d=5$” and “$\text {IS}=640^2$, $d=5$” with model width $c_0=96$), we notice a slight drop in the mean IoU. The reason is the large number of parameters these models have with approximately 280 million and the small dataset we used for training.

### Multi-axes prediction fusing

Table 2 shows the results for processing the 3D volume with a simple 2D slice-by-slice approach and the additional results of label fusing. For the baseline (where the volume is only sliced in one direction), we see that $S_{rc}$ achieved a slightly higher mean IoU with 0.9022 than $S_{pc}$ and $S_{pr}$ with 0.8973 and 0.8971, respectively. The most challenging class is the “degradation layer” where all three have a lower mean IoU with 0.7931 to 0.8016. The other classes “bone” and “residual material” are substantially higher with 0.9672 to 0.9683 and 0.9365 to 0.9310, respectively.

Fusing the information of all three baseline slices (i.e., shown as “3-planes” in Table 2) with soft- and majority voting helps to improve the overall mean IoU (i.e., 0.9070 and 0.9048, respectively) and the IoU for each class. Here, soft voting performs slightly better than MV. The inclusion of our proposed additional slices reduced the overall mean IoU from 0.9070 to 0.9057 for soft voting when compared to “3-planes”. Nevertheless, the IoU for the class “degradation layer” increased to 0.8133.

**Figure 4:**
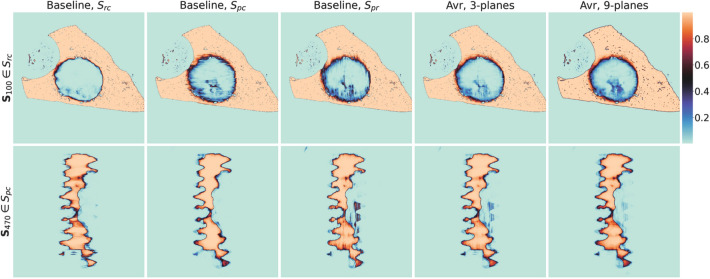
Probability results for processing the 3D volume slice-by-slice and the proposed soft voting fusing method. Here, we show ${\mathbf {S}}_{100} \in S_{rc}$ and ${\mathbf {S}}_{470} \in S_{pc}$ (in the first and second row, respectively) of the test sample 1. For ${\mathbf {S}}_{100} \in S_{rc}$, each image shows the probability output for “bone” of our best model without conversion to a final segmentation. For ${\mathbf {S}}_{470} \in S_{pc}$, we show the probability output for “residual material”. From left to right: “Baseline, rc”, “Baseline, pc”, “Baseline, pr”, “Avr, 3-planes”, and “Avr, 9-planes”. A high value indicates that this area is most likely “bone” or “degradation layer” for ${\mathbf {S}}_{100} \in S_{rc}$ and ${\mathbf {S}}_{470} \in S_{pc}$, respectively.

Figure 4 shows the effect of the slice-by-slice processing. We can see that using only slices from one direction introduces inconsistency artifacts (i.e., striking lines in horizontal and vertical direction for $S_{pc}$ and $S_{pr}$, respectively) in the direction the 3D volume was sliced. For the first row with ${\mathbf {S}}_{99} \in S_{rc}$, the artifacts are visible for $S_{pc}$ and $S_{pr}$, but not for $S_{rc}$. The inconsistency are not shown for $S_{rc}$ because the example shows an image in the same slicing direction. For “3-planes” and “9-planes”, we observe that these striking artifacts are increasingly reduced by multi-axes prediction fusing. We observe the same for the second example in the second row where ${\mathbf {S}}_{470} \in S_{pc}$ is shown. Here, the artifacts are also visible in horizontal and vertical direction for $S_{rc}$ and $S_{pr}$, respectively.

### Visual and quantitative analysis of segmentation

**Figure 5:**
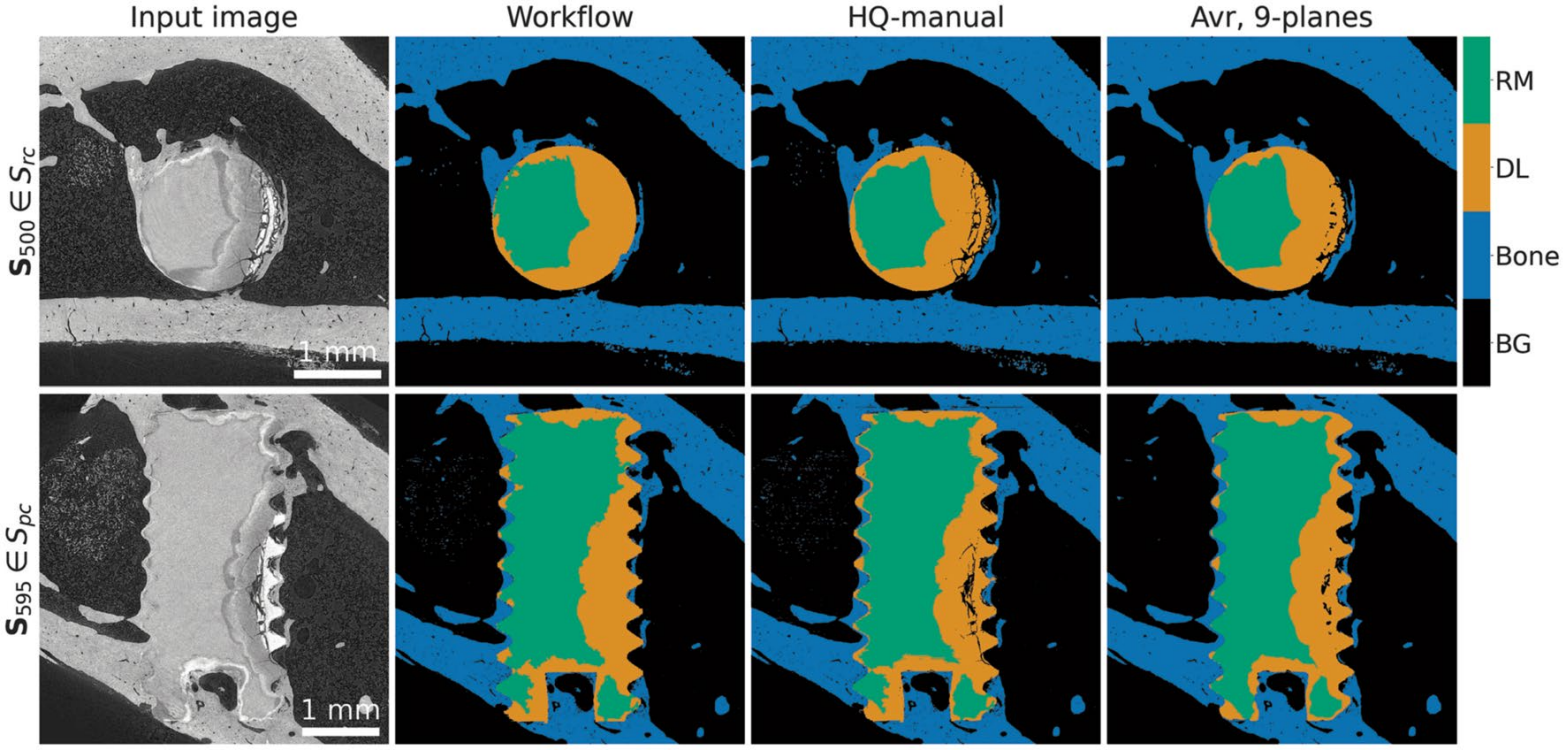
Comparison between different types of segmentation results—high quality (manual), workflow, and machine learning (Avr., 9-planes). We show ${\mathbf {S}}_{500} \in S_{rc}$ and ${\mathbf {S}}_{595} \in S_{pc}$ (in the first and second row, respectively) of test sample 1. For the segmentation results, the images are colored based on the corresponding label: residual material (RM), degradation layer (DL), bone, and background (BG).

Figure 5 shows representative slices of the image data and the corresponding segmented data sets for visual comparison. The quality of the segmentation is assessed by crack appearance in the degradation layer, the overall smoothness and accuracy in the bone structure. The workflow segmentation does not give optimal results. In many regions residual material and degradation layer are incorrectly detected because of the similarities in grayscale and inaccuracies in matching pixels to proper label. Moreover, the workflow segmentation does not include cracks in the degradation layer which has an effect on the quantification of the performance and on the training of the U-net.

Furthermore, we compare the WF and ML segmentation to the HQ segmentation based on three important parameters: DR, BIC, and BV/TV (see Eqs. 1, 2, and 3 ). The quantified parameters based on these segmentations are given in the Table 3. We consider HQ segmentation as the reference, because it was manually corrected with the highest precision.

First, we consider the DR parameter. Values from WF and ML segmentation vary by less than 10% from the reference one. The only variable which influences this result is the volume of residual material. It is the highest for HQ, because after manual corrections more pixels were included into a degradation layer—less residual material means higher degradation rate. Next parameter, the BIC, is dependent on the contact area between the combination of residual material and degradation layer and the bone label. Here, we observe noticeable differences in some BIC values, especially for the sample 3. The reason for this is the degradation layer, which is heavily fragmented and full of cracks at the top and the bottom of the implant. Those cracks increase the surface area *a* substantially for the BIC calculation and, therefore, the BIC is reduced. Here, ML segmentation is better than WF segmentation because the model segments larger cracks while the WF segmentation does not consider cracks. Parameter BV/TV shows the smallest deviation (up to 4%) because it is only dependent on the bone and background labels. The segmentation of the bone is straightforward because of the good contrast with the background.

For visualization of aforementioned differences, we present comparison of the segmentation quality for each sample in Supplementary Figs. S2, S3, and S4.

## Discussion and conclusion

We presented a systematic evaluation of distinct design decisions for the semantic segmentation of SRμCT images of bone-implants using a U-net. Scaling the baseline U-net by a single hyper-parameter depth, width or input size, we observed an improvement in our results. Nevertheless, the compound scaling of all three parameters achieved the overall best mean $\text {IoU} = 0.906$. We also noticed a drop in the performance for the very large models with approximately 280 millions parameters. This is due to the fact that our training data set with 14 samples is very small and therefore the model started to overfit.

Our experiments for multi-axes prediction fusing showed that it is beneficial to include multiple slicing directions of the 3D volume. Furthermore, we showed that soft voting is superior to majority voting. The in-depth analysis of the prediction probabilities showed that adding more slicing direction reduces striking artifacts. Although the numerical metric (i.e., IoU) showed no improvement for the average and only a minor improvement for the class “residual material”, we found that adding more slice directions smoothed the segmentation boundary. In the subsequent quantitative analysis, the boundary has a large influence on the measurements, so a smooth boundary is desired.

The quantitative analysis and visual inspection showed that our best performing ML model is better than the current workflow segmentation method, which is noteworthy since the network was only trained on the WF segmentation data. The WF segmentation often failed to segment small and larger cracks in the degradation layer. The ML model, on the other hand, is at least capable of segmenting larger cracks. Unfortunately, small cracks are also not segmented by the model. The problem might be the noisy training data from the WF segmentation because most of the cracks are not correctly segmented. Therefore, it is very hard to learn such a feature for the model.

Overall the ML segmentation results deviate less from the HQ segmentation, as compared to WF segmentation. Consequently, the ML segmentation provides a more reliable segmentation result for the quantification of osseointegration and degradation parameters. Although the ML segmentation does not provide perfect results, it does improve comparability and eliminates human bias. In addition, the time required to obtain a segmentation result was reduced from four days to 20 min for WF segmentation and ML segmentation, respectively. Nevertheless, to achieve even better results we must consider correcting the ML segmentation. This step can be performed automatically by smoothing the labels and automatic thresholding. These additional steps include the detection of cracks in the degradation layer and cannulations (or small channels in the bone) in the bone.

In future work, we suggest to further investigate the problem of crack segmentation. Here, a second model trained exclusively for such fine-grained features should be useful. This training should be further improved by implementing a crack simulation for more complex data augmentation. Finally, we also suggest to explore other loss function which can help to smooth the boundaries of the segmentation results.

**Table 1:** Synchrotron radiation microtomography dataset characteristics.

		**Training dataset**	**Testing dataset**
3D Samples		14	3
2D Images		47,600	–
**Alloy type**			
Mg-5Gd		4 (29%)	3 (100%)
Mg-10Gd		10 (71%)	–
**Beamline**			
IBL		9 (64%)	1 (33%)
I13-2		5 (36%)	2 (66%)

For training, each sample is sliced into 2D images based on the three main axes. For testing, the number of 2D images changes based on the multi-axes fusing method.

**Table 2:** Mean Intersection over Union (IoU) and the standard error results for different 3D information fusing methods.

**Method**	**Bone**	**Degradation layer**	**Residual material**	**Avr**
Baseline, $S_{rc}$	$96.83 \pm 0.14$	$80.16 \pm 0.39$	$93.65 \pm 0.15$	$90.22 \pm 0.21$
Baseline, $S_{pc}$	$96.72 \pm 0.08$	$79.31 \pm 0.26$	$93.16 \pm 0.06$	$89.73 \pm 0.09$
Baseline, $S_{pr}$	$96.72 \pm 0.11$	$79.32 \pm 0.29$	$93.10 \pm 0.07$	$89.71 \pm 0.12$
Avr, 3-planes	$\mathbf {97.01} \pm \mathbf {0.10}$	$81.26 \pm 0.34$	$93.83 \pm 0.06$	$\mathbf {90.70} \pm \mathbf {0.12}$
MV, 3-planes	$96.95 \pm 0.10$	$80.76 \pm 0.32$	$93.73 \pm 0.06$	$90.48 \pm 0.11$
Avr, 9-planes	$96.38 \pm 0.12$	$\mathbf {81.33} \pm \mathbf {0.33}$	$\mathbf {93.99} \pm \mathbf {0.08}$	$90.57 \pm 0.11$
MV, 9-planes	$96.32 \pm 0.12$	$81.07 \pm 0.31$	$93.94 \pm 0.08$	$90.44 \pm 0.10$

Bold text for each column emphasizes the overall highest mean IoU value. All values are scaled by 100 for convenience.

**Table 3:** Comparison of the quantified parameters for each type of segmentation.

**Parameters**	**Sample ID**	**WF**	**HQ**	**ML**
DR [mm/year]	1	0.252 (−3%)	**0.261**	0.243 (−7%)
	2	0.205 (−2%)	**0.209**	0.208 (−)
	3	0.417 (−10%)	**0.462**	0.436 (−6%)
BIC [%]	1	62.94 (−)	**62.97**	70.44 (+12%)
	2	81.07 (+1%)	**80.22**	80.30 (−)
	3	60.13 (+33%)	**45.14**	51.61 (+14%)
BV/TV [%]	1	47.88 (−1%)	**48.14**	48.45 (+1%)
	2	55.23 (+1%)	**54.93**	54.67 (−)
	3	41.25 (+4%)	**39.64**	41.25 (+4%)

We consider high quality (HQ) segmentation as the reference (in bold). Percentage values in brackets represent the relative differences between workflow (WF) and machine learning (ML) segmentation compared to the HQ segmentation. (−) means that the difference was less than 1%.

## Supplementary Information

Supplementary Information 1.

## Data Availability

The datasets generated during and/or analyzed during the current study are available from the corresponding author on reasonable request.
