# Supplementary material for: Scaling the U-net: segmentation of biodegradable bone implants in high-resolution synchrotron radiation microtomograms
Source: Sci Rep. 2021 Dec 20;11:24237. doi: 10.1038/s41598-021-03542-y (PMC8688506; doi:10.1038/s41598-021-03542-y)
Supplement: Supplementary file 1 — Supplementary Information 1. [file 41598_2021_3542_MOESM1_ESM.pdf]

# Scaling the U-net: Segmentation of biodegradable bone implants in high-resolution synchrotron radiation microtomograms

**Ivo M. Baltruschat[1,+,*], Hanna Ćwieka[2,+], Diana Krüger[2], Berit Zeller-Plumhoff[2], Frank Schlünzen[1], Regine Willumeit-Römer[2], Julian Moosmann[3,*], and Philipp Heuser[1,4]**

[1]Deutsches Elektronen-Synchrotron DESY, Notkestr. 85, 22607 Hamburg, Germany
[2]Institute of Metallic Biomaterials, Helmholtz-Zentrum hereon GmbH, 21502 Geesthacht, Germany
[3]Institute of Materials Physics, Helmholtz-Zentrum hereon GmbH, 21502 Geesthacht, Germany
[4]Helmholtz Imaging Platform, Deutsches Elektronen-Synchrotron DESY, Notkestr. 85, 22607 Hamburg, Germany
[*]ivo.baltruschat@desy.de
[*]julian.moosmann@hereon.de
[+]these authors contributed equally to this work

## Supplementary Information

## Illustration of the segmentation problem

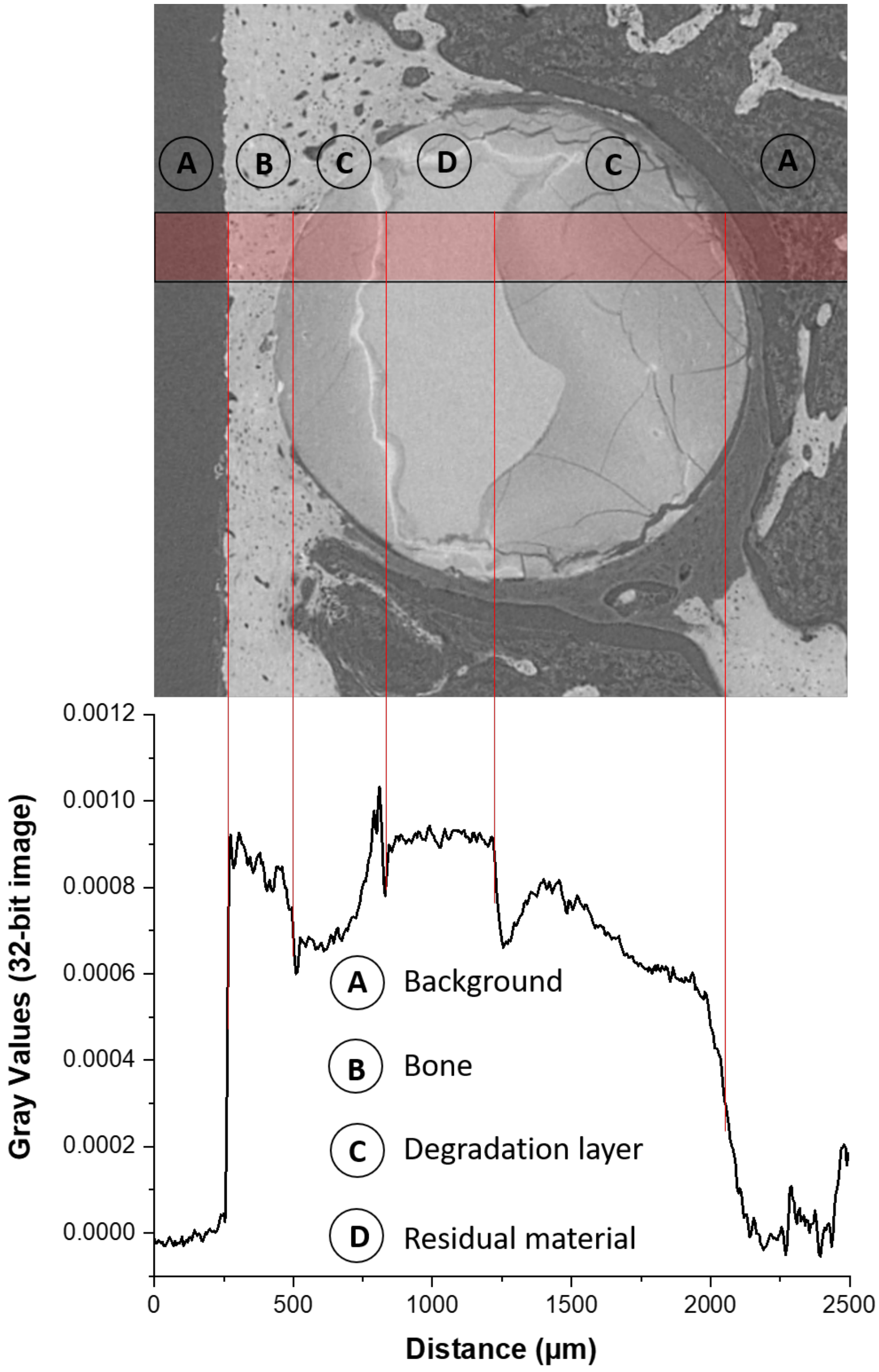


**Figure S1.** Gray value for each section: background, bone, degradation layer and residual material.

## Workflow segmentation

The procedure for workflow segmentation is as follows:

1. Import the reference and the preimplantation screw into Avizo with the correct pixel size for both data sets.
2. Register and resample the preimplantation screw onto the reference screw.
3. Import the corresponding explant with the correct pixel size, register and resample it on the preimplantation screw. As a result, we obtain 1200x1200x1000 lattice and 5 μm pixel size of preimplantation screw and explant data set. All next steps are performed on the registered and resampled data sets.
4. Convert the preimplantation screw into a label and attach it to the registered and resampled explant. As a result, the preimplantation screw is oriented in the same way as the explant screw.
5. Manually correct the mistakes where the preimplantation screw does not match the borders of the explant screw. As a result, we obtain segmented label of overall explant screw (residual material + degradation layer).
6. Create labels for residual material and degradation layer on the segmented overall explant screw and perform watershed segmentation. As a result, we obtain segmented labels of residual material and degradation layer.
7. Perform the automatic thresholding on the explant data set with locked label of explant screw. As a result, we obtain segmented label of bone.

## Baseline U-net architecture

**Table S1.** (a) and (b) show the baseline encoder and decoder architecture, respectively. “Conv3x3BNAct“ refers to a convolution with a $3 \times 3$ kernel, batch normalization (BN), and activation function (i.e., in our case Mish). The columns “Resolution” and “#Channels” each represent the output dimensions of this operator. “#Layers” are the number of consecutive operator. “ConvTrans2x2” is a transposed convolution with a $2 \times 2$ kernel to upsample the image.

**(a)**

Encoder

| Block | Operator | Resolution [pixels] | #Channels | #Layers |
|---|---|---|---|---|
| 1 | Conv3x3BNAct | $384 \times 384$ | 32 | 2 |
| | MaxPooling2x2 | $192 \times 192$ | 32 | 1 |
| 2 | Conv3x3BNAct | $192 \times 192$ | 64 | 2 |
| | MaxPooling2x2 | $96 \times 96$ | 64 | 1 |
| 3 | Conv3x3BNAct | $96 \times 96$ | 128 | 2 |
| | MaxPooling2x2 | $48 \times 48$ | 128 | 1 |
| 4 | Conv3x3BNAct | $48 \times 48$ | 256 | 2 |

**(b)**

Decoder

| Block | Operator | Resolution | #Channels | #Layers |
|---|---|---|---|---|
| | ConvTrans2x2 | $96 \times 96$ | 256 | 1 |
| 5 | Conv3x3BNAct | $96 \times 96$ | 128 | 2 |
| | ConvTrans2x2 | $192 \times 192$ | 128 | 1 |
| 6 | Conv3x3BNAct | $192 \times 192$ | 64 | 2 |
| | ConvTrans2x2 | $384 \times 384$ | 64 | 1 |
| 7 | Conv3x3BNAct | $384 \times 384$ | 32 | 2 |
| | Conv1x1Softmax | $384 \times 384$ | 4 | 1 |

## Bone to implant contact

Bone to implant contact (BIC) is a parameter describing how much of the degraded implant is in contact with mineralized bone and is quantified by dividing the surface area being in contact with bone the overall surface area of the implant.

$$\text{BIC} = \frac{b}{a}, \tag{1}$$

where $b$ is the total number of boundary voxel between the implant and bone and $a$ is the surface area of the implant.

**Table S2.** Contact surface area $b$, surface area $a$ and BIC for each sample obtained with different segmentation techniques

| Sample | Segmentation | $b$ [mm$^2$] | $a$ [mm$^2$] | BIC [%] |
|---|---|---|---|---|
| Sample 1 | WF | 34.36 | 54.58 | **62.94** |
| | HQ | 35.53 | 56.43 | **62.97** |
| | ML | 38.83 | 55.12 | **70.44** |
| Sample 2 | WF | 42.01 | 51.82 | **81.07** |
| | HQ | 45.93 | 57.25 | **80.22** |
| | ML | 47.73 | 59.44 | **80.30** |
| Sample 3 | WF | 36.68 | 60.99 | **60.13** |
| | HQ | 30.62 | 67.82 | **45.14** |
| | ML | 29.85 | 57.83 | **51.61** |

## Result example for sample 1

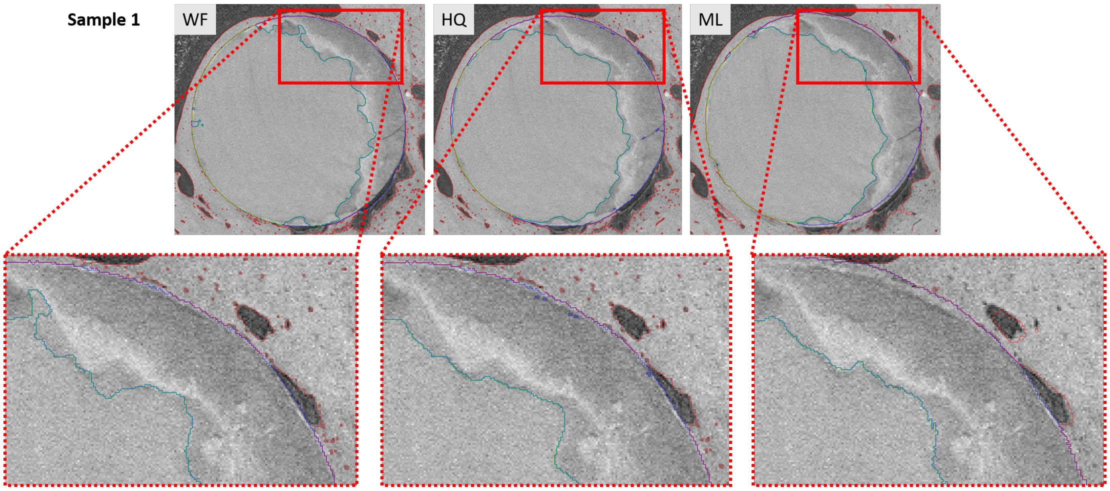


**Figure S2.** Sample 1. Comparison of the quality of different types of segmentation (shown as outline): semi-automatic workflow (WF), high-quality (HQ), and machine learning (ML). HQ is the reference segmentation.

## Result example for sample 2

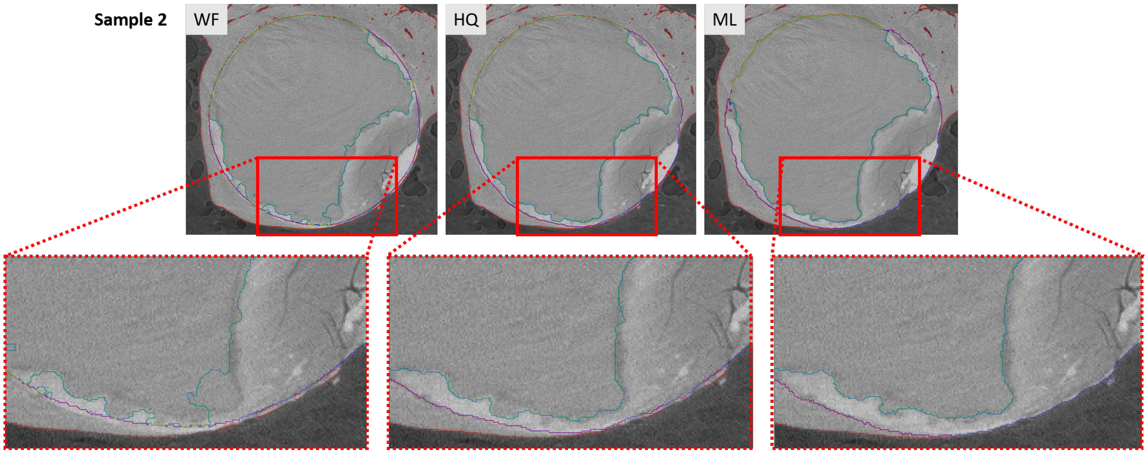


**Figure S3.** Sample 2. Comparison of the quality of different types of segmentation (shown as outline): semi-automatic workflow (WF), high-quality (HQ), and machine learning (ML). HQ is the reference segmentation.

## Result example for sample 3

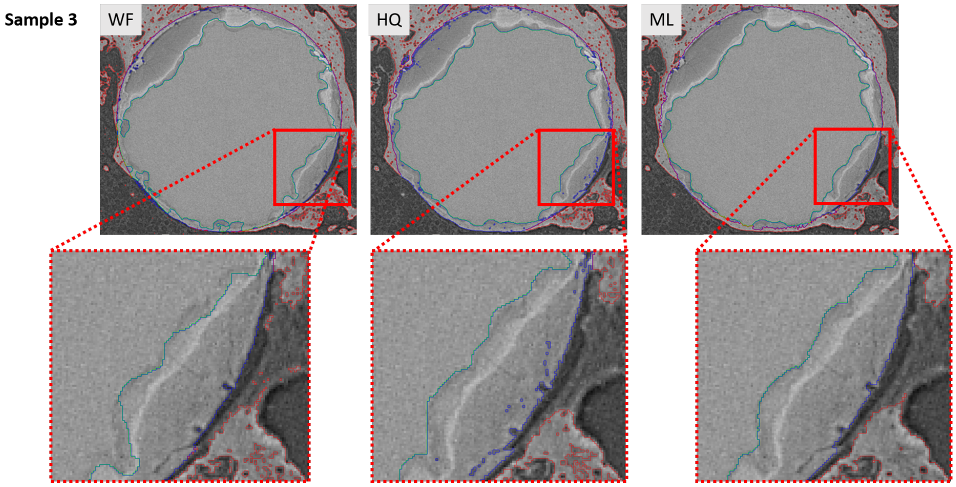


**Figure S4.** Sample 3. Comparison of the quality of different types of segmentation (shown as outline): semi-automatic workflow (WF), high-quality (HQ), and machine learning (ML). HQ is the reference segmentation.
